# Investigating employment anxiety among pre-service Chinese teachers in Taiwan, China: influencing factors and implications

**DOI:** 10.3389/fpsyg.2026.1797490

**Published:** 2026-04-01

**Authors:** Ling Shen, Xianghua Yan

**Affiliations:** Chinese Language and Culture College, Huaqiao University, Xiamen, China

**Keywords:** anxiety performance, employment anxiety, external influence, pre-service Chinese teachers, psychological construction, self-cognition

## Abstract

In the context of the global importance of the Chinese language and the post -pandemic employment difficulties, this research delves into the employment anxiety of pre - service Chinese teachers in Taiwan, China, examining its influencing factors and implicationsit. It employs questionnaire surveys (*N* = 241) and interviews (*N* = 16) to analyze variables such as self - perception, psychological constitution, external impacts, and manifestations of anxiety. Statistical analysis reveals significant correlations. Employment anxiety is positively correlated with neuroticism and career planning, and negatively correlated with self - regulation and family income. Group disparities are identified: students from public schools exhibit higher levels of self -perception anxiety, female students display stronger psychological constitution anxiety, and students from middle - income families report the highest anxiety levels. The key influencing factors include relevant internship experience (which mitigates anxiety), language proficiency (students proficient in English and a minority language show the lowest anxiety), and low policy awareness (only 26.14% are familiar with industry policies). The combined explanatory power of self - perception, psychological constitution, and external impacts on anxiety amounts to 75.25%.This study underscores the significance of psychological resilience and career guidance in alleviating anxiety. The implications suggest strengthening pre - service teacher training by providing targeted psychological support, practical career strategies, and policy education to enhance employability in the competitive educational market.

## 1 Introduction

### 1.1 Research background

In recent years, Chinese has become a significant global language. Before 2020, most graduates from Chinese language and literature programs in Taiwan, China, taught abroad or locally. However, due to the pandemic, they were reluctant to teach overseas. Although online teaching has low entry barriers, many experienced teachers moved their courses online. Graduates, lacking teaching experience, have trouble passing online educational institution interviews or get low hourly pay, leading to few positions and high unemployment. Statistics from the 104 Job Bank on May 29, 2023, show that science-related graduates in Taiwan, China, generally earn more than humanities graduates. Comparing employment three years after graduation, master’s graduates from Chinese language and literature programs and software-related science and engineering programs have monthly salaries of NT$55,031 and NT$78,610, respectively. The gap between humanities and science positions is large, making Chinese language and literature graduates particularly anxious.

From the perspective of psychological research, employment anxiety is the tension and uncertainty students face in job-seeking. It often shows as a lack of positive outlook and low morale. These emotions can cause psychological and physiological changes, significantly affecting students’ daily lives. Thus, solving college students’ employment-related psychological problems is a basic responsibility of higher education institutions and crucial for the smooth implementation of employment initiatives.

### 1.2 Literature review

Employment anxiety is an anxiety response associated with specific life events (employment), which may coexist with broader mental health issues. For instance, studies have shown that COVID-19 phobia as a specific fear can significantly induce occupational anxiety. This implies that major social crises (such as global pandemics) as external stressors may exacerbate individuals’ concerns about job stability and security. Consequently, pervasive social anxiety manifests as anxiety in the employment domain (Mahmud et al., 2021).

Employment anxiety can be viewed as a process of re-evaluating and perceiving loss of potential resources during the transition from academic environments (including academic achievements and psychological resilience) to occupational environments (transforming into resources such as job stability and career development) (Yang et al., 2025). In contrast, job anxiety or occupational anxiety primarily refers to anxiety experienced by employed individuals due to workplace environmental factors (such as workplace harassment, job insecurity, technical pressure, or excessive competence of colleagues). In certain professional fields (such as nursing and STEM fields), issues like competitive career development paths, high standards, or gender differences are particularly prominent. For example, nursing interns often face challenges related to occupational anxiety (Zhang J. et al., 2025; Zhang S. et al., 2025), while women in STEM fields may experience specific occupational anxiety due to perceiving their qualifications as excessive (Khan et al., 2024). Despite their different backgrounds, both share similar psychological mechanisms. For instance, the resource conservation theory is often used to explain these phenomena: anxiety arises when individuals perceive threats or losses of resources (such as time, energy, or job security) (Wang G. et al., 2025; Wang L. Y. et al., 2025; Nauman et al., 2019; Zhang et al., 2023; Samma et al., 2020).

Employment anxiety is not a single-dimensional emotion, but a complex psychological construct closely related to an individual’s perception of employment risks, competitive environment, self-assessment of abilities, and future career development (Liang and Zhai, 2025; Söner and Yılmaz, 2025).

Employment anxiety is not an isolated phenomenon, but dynamically balanced with multiple positive psychological traits. Among these, psychological resilience (or stress resistance) has been repeatedly confirmed as a key protective factor in mitigating employment anxiety (Yang et al., 2025; Wang G. et al., 2025; Wang L. Y. et al., 2025). Individuals with higher psychological resilience demonstrate stronger stress adaptation and post-failure recovery capabilities, resulting in significantly lower anxiety levels when facing academic pressure and employment uncertainty (Yang et al., 2025).

Anxiety levels fluctuate with job-seeking progress, external economic conditions (e.g., the post-pandemic era) (Yang et al., 2025), technological advancements (e.g., AI applications) (Liang and Zhai, 2025; Zhang J. et al., 2025; Zhang S. et al., 2025), and personal growth phases (e.g., graduation preparation) (Li et al., 2025). Research indicates that clear career goals, self-awareness, high self-esteem (both explicit and implicit), and goal-oriented learning can effectively alleviate employment anxiety or mitigate its negative effects (Wang G. et al., 2025; Wang L. Y. et al., 2025; Liang and Zhai, 2025). These factors collectively form an individual’s psychological toolkit for navigating employment challenges.

In summary, employment anxiety primarily emerges during the pre-employment phase, particularly in the transition from school to the workplace or during job-seeking processes (Yang et al., 2025; Li et al., 2025). Employment anxiety is a multidimensional and dynamically evolving psychological state, defined as a persistent negative emotional response experienced by individuals during career transitions or job searches due to perceived internal and external pressures and threats. While distinct from general anxiety and work-related anxiety, it is interconnected with both, serving as both a psychological consequence of stressful events and a mediating mechanism influencing other cognitive and behavioral processes. In contemporary contexts, its manifestations are significantly influenced by technological advancements and digital social environments, giving rise to new characteristics such as the “reinforcement paradox” (Li et al., 2025). Understanding employment anxiety requires considering both its triggering factors (e.g., academic pressure, social comparison, technological anxiety) (Yang et al., 2025; Li et al., 2025; Mahmud et al., 2021; Liang and Zhai, 2025) and protective factors (e.g., psychological resilience, self-concept, social support efficacy) (Yang et al., 2025; Wang G. et al., 2025; Wang L. Y. et al., 2025), which provide clear theoretical targets for subsequent intervention research.

Against the backdrop of global economic volatility, industrial restructuring, and accelerated technological transformation, labor market uncertainties have significantly intensified. Particularly in the post-pandemic era, uneven economic recovery has further exacerbated employment pressures for groups like university graduates (Yang et al., 2025). This macroeconomic instability directly impacts individuals, emerging as a major external stressor contributing to employment anxiety.

Since 2020, scholars across the globe have conducted research on the employment anxiety of college students during the pandemic, with a focus on the employment environment and personal emotional regulation. Although foreign researchers have not explored the pre-pandemic anxiety levels or the factors contributing to employment anxiety, their remarkable achievements in campus management and employment market research provide valuable policy implications.

This study conducts an investigation on four distinctive universities in Taiwan, China, which specialize in Chinese language education. These universities include the top normal university in the region, the first institution to establish a Chinese language department in Taiwan, China, the only public university in Taiwan’s outer islands, and the sole foreign language and trade university. Despite the differences in their founding backgrounds, educational goals, and curriculum focuses, all four institutions maintain the leadership’s commitment to promoting the all-round development of students through academic programs.

The research comprehensively examines the employment anxiety among Taiwanese graduates of the 2023–2024 cohort from four dimensions: the perspectives of students, the institutional curriculum design, and the support from family and social policies. Ultimately, it proposes practical solutions to alleviate anxiety, promote the harmonious development of universities, and offer references for domestic research on graduate anxiety management.

## 2 Method

### 2.1 Questionnaire design process

Although a significant amount of research on employment anxiety among college students has been carried out globally, there is a dearth of studies concentrating on this issue among graduates from the Chinese language and literature departments in Taiwan, China. To ensure the scientific rigor and precision of the survey, substantial efforts were made in the development of the questionnaire concerning employment anxiety and its influencing factors for students in this department.

Initially, an initial version of the questionnaire was designed via an analysis of the department’s curriculum structure and employment trends. Simultaneously, insights from relevant studies on family background, career planning, and factors influencing employment anxiety were incorporated to formulate the core items.

Subsequently, a pilot study was conducted by administering the draft questionnaire to a small sample of participants. Questions with ambiguous formulations were removed, and the questionnaire was refined in light of the feedback obtained.

Prior to the formal survey, the department sought evaluations from faculty members, which resulted in further improvements in the quality of the questionnaire.

Finally, a standardized survey instrument was compiled and distributed for data collection, thus establishing the basis for subsequent empirical research analysis.

### 2.2 Variable design of the questionnaire

Based on the research objectives and the outcomes of factor analysis, and in accordance with the “influencing factors-outcome” logical framework, this study categorizes variables into two types: influencing factors (internal + external) and anxiety manifestation. The internal factors encompass self-cognition and psychological structuring. Self-cognition pertains to an individual’s subjective evaluation of their competencies, skills, and career advancement, which reflects students’ core beliefs regarding their ability to proficiently teach Chinese as a second language. Psychological structuring signifies an individual’s mental preparedness for employment, including adaptation to identity transformation, salary expectations, and stress-coping capacity. External influence represent the objective environmental effects on employment anxiety, including school support, family support, the impacts of the pandemic, and the policy environment. Employment anxiety manifestation serves as the dependent variable, representing the specific behavioral or emotional manifestations of employment anxiety resulting from the influence of internal factors, such as insomnia, a sense of urgency, and interpersonal effects. Self-cognition and psychological structuring correspond to individual-level psychological mechanisms (self-efficacy, career efficacy), while external influence corresponds to environmental-level stressors (e.g., social support, policy environment). These three factors jointly affect the levels of employment anxiety, ultimately manifested through anxiety manifestation. The specific items for each variable are presented in Table 1.

**Table 1:** Specific measurement indicators for each variable.

Variable name	Serial number	Measurement indicators
Self-cognition	ZW1	I do not have high expectations for the future development of my major.
	ZW2	I feel unable to manage interpersonal relationships after entering the workforce.
	ZW3	I believe my current skills are insufficient to support future teaching responsibilities.
	ZW4	I am feeling unable to excel in future work.
	ZW5	I do not think I can find job information accurately.
Psychological construction	XL1	I do not prepared for the transition of social identity.
	XL2	I do not think my salary after starting work will meet my expectations.
	XL3	I’m feeling that one’s work falls short of parental expectations.
	XL4	It’s hard to accept getting rejected multiple times when you keep sending out your resume.
	XL5	I believe that one cannot find a satisfactory job with their current educational background.
External influence	WJ1	I feel that the career guidance offered on campus is of no help to me.
	WJ2	I fear the pandemic’s impact on employment will severely affect me.
	WJ3	I do not think my family will help me get a job.
	WJ4	I believe that campus experience alone is insufficient to meet the demands of post-employment work.
	WJ5	I do not think internships in my field can help with employment.
Anxiety performance	JV1	I believe that job-hunting anxiety has severely impacted my daily life.
	JV2	I feel like I’ll be tossing and turning before the actual interview.
	JV3	The sense of urgency intensifies as graduation approaches.
	JV4	Anxiety persists even with thorough interview preparation.
	JV5	The anxiety about job hunting has already affected people around me.

The measurement indicators for the self- cognition, psychological construction,external influence and anxiety performance variable are mainly based on the study by Zhao and Li (2022), Li Q. J. (2019), Xu T. (2022) and Pei W. Q. et al. (2022) respectively.

### 2.3 Survey questionnaire content

A questionnaire, constructed on the basis of the Likert five-point scale, was disseminated to students majoring in Chinese language teaching from the 2023 and 2024 cohorts at four universities in Taiwan, China. The questionnaire is composed of four sections.

Section 1 is devoted to gathering fundamental student information, which encompasses school type, gender, and nine other items.

Section 2 assesses employment readiness, containing seven inquiries regarding future job-seeking regions.

Section 3 presents a core scale with four dimensions: self-cognition, psychological construction, external influence, and anxiety performance. To accurately gauge anxiety levels among students of different grades, the five-point scale allows respondents to select the most suitable criteria. In this scale, higher scores indicate lower levels of agreement, and there are a total of 20 questions.

Section 4 provides anxiety management solutions for students’ reference in the study.

### 2.4 Interview outline design

To ensure the accuracy of preliminary survey results, an interview outline was developed during the design of the questionnaire on employment anxiety and influencing factors among students of Chinese language teaching in Taiwan, China. The process began with supplementing the interview content through questionnaire items to initially design the outline, and then refining the questions by referring to relevant interview studies. Subsequently, a pre-enrollment survey was conducted to confirm with the participating students, and items not closely related to employment anxiety were removed to form a comprehensive interview outline.

Based on the questionnaire, this interview outline supplemented data from students at four universities, structured into three sections, each containing five questions. The first section explores students’ pre-enrollment perceptions and plans for their majors, including differences in professional experiences and prospects before and after enrollment. The second section focuses on disparities in the teaching environment during study and internships, covering teaching languages and professional confidence. The third section investigates employment anxiety at graduation and advice from alumni, including anxiety-related events and suggestions for various departments.

### 2.5 Overview of questionnaire and interview implementation

The survey research employs a purposive sampling framework. Regarding institutional selection, four Chinese language teaching departments in Taiwan were chosen, including both public and private institutions, to ensure the representativeness of diverse educational backgrounds and reflect the student diversity within these departments. A comparative analysis of the institutional establishment contexts and teaching objectives reveals disparities in curriculum design and student anxiety levels between public and private schools. Using proportional sampling based on graduation years, the study focuses on the graduates of 2023 and 2024, aiming to conduct year-by-year comparisons to analyze the trends in employment anxiety. The survey adopts a word-of-mouth promotion model for questionnaire dissemination, collects data via Google Forms, and optimizes the questionnaire design through pilot testing and expert evaluation to enhance data quality.

The online survey was conducted from April 15 to May 1, 2023. A total of 255 questionnaires were disseminated, and all were retrieved. To ensure the accuracy and comprehensiveness of the results, statistically invalid responses were excluded. Specifically, 14 invalid questionnaires were removed, yielding 241 valid responses, with an effective response rate of 94.5%.

This interview study randomly selected one male and one female student from each school, representing the student cohorts of 2023 and 2024. The interview content was organized into three aspects: factors considered in pre-enrollment decision-making, major-related cognitions during the study process, and manifestations of employment anxiety upon graduation, with the aim of supplementing the questionnaire survey. Due to time and transportation constraints, the interviews were conducted from November 15 to December 31, 2023, through a combination of online meetings and face-to-face interviews, involving 16 participants. To ensure the accuracy and comprehensiveness of the results, the interviewers provided meals during the interview sessions and offered remuneration to students who participated in the online interviews.

## 3 Results

### 3.1 Reliability and validity analysis of the questionnaire

This study employed SPSS 25.0 to conduct reliability, validity, and factor analyses on the questionnaire data. Factor analysis, a widely used statistical tool for assessing structural validity, aims to identify the underlying structural factors, verify the structural validity of the survey scales, explore the components of college students’ employment anxiety, and select key parameters. Reliability testing evaluates the credibility or stability of the questionnaire, typically measured by the Cronbach’s Alpha coefficient. Higher values of this coefficient indicate greater reliability. Generally, coefficients above 0.9 signify high reliability; those in the range of 0.8–0.9 suggest good data quality; values between 0.7 and 0.8 are considered acceptable; and coefficients below 0.7 imply the need for questionnaire revision. The indicators designed in this research included self-cognition, psychological construction, external influence, and anxiety manifestation. The data in Table 2 shows that the Cronbach’s Alpha coefficients of all dimensions exceeded 0.7, meeting psychometric standards, indicating overall satisfactory reliability and suitability for further investigation.

**Table 2:** Reliability of each dimension.

Dimension	Cronbach’s α coefficient	Number of items	Reliability evaluation
Self-cognition	0.82	5	Good (α > 0.8)
External influence	0.78	5	Good (0.7 < α < 0.8)
Psychological construction	0.76	5	Acceptable (0.7 < α < 0.8)
Anxiety performance	0.81	5	Good (α > 0.8)
Total	0.89	20	Excellent (α > 0.85)

This study employed the KMO test and Bartlett’s Sphericity test to validate the sample and data. Generally, the greater the number of common factors among variables, the higher the KMO value, which signifies the suitability for factor analysis; conversely, lower values suggest inappropriateness. A KMO value exceeding 0.9 is highly suitable, a value within the range of 0.8–0.9 is appropriate, 0.7–0.8 is acceptable, 0.6–0.7 is less favorable, and a value below 0.6 is highly unsuitable. As shown in Table 3, the KMO value of this five-item scale is 0.862, falling within the 0.8–0.9 range, indicating strong correlations among items and facilitating in-depth investigation. The Bartlett’s Sphericity test yielded a parameter of 10310.362, with 190 degrees of freedom and a significance level of 0.000 (*p* < 0.01), confirming the non-uniform distribution of the data and its suitability for subsequent analysis.

**Table 3:** Results of KMO and Bartlett’s Sphericity tests.

KMO and Bartlett’s Sphericity test		
KMO sample adequacy measure		0.862
Bartlett’s Sphericity	Approximate Chi-square	10310.362
	Degrees of freedom	190
	Sig.	0.000

### 3.2 Exploratory factor analysis

Factor analysis is employed to identify the intrinsic structure of multivariate observations and conduct dimensionality reduction. It integrates related factors into a unified categorization to generate principal component factors, thus consolidating complex variables into a limited number of core factors. In comparison with traditional methods, principal component analysis avoids problems such as outliers, equidistant values, linear relationships, multivariate normal distribution, and orthogonality. This method facilitates a thorough exploration of variable correlations, and at this juncture, principal component analysis will be utilized to extract common factors.

As shown in Table 4, the principal component analysis (PCA) identified four factors with eigenvalues exceeding 1. Before rotation, the initial eigenvalues were 9.349, 2.092, 1.634, and 1.223 respectively, while the eigenvalue of the fifth factor was less than 1. After rotation, the new eigenvalues were 4.112, 3.728, 3.347, and 3.112. These four common factors contribute 75.252% to the total variance, exceeding 60%, which implies strong structural validity. This demonstrates the model’s ability to accurately capture the employment anxiety data of Chinese language teaching majors in Taiwan, China.

**Table 4:** Common factor extraction.

Component	Initial eigenvalues			Extracted sum of squared loadings			Rotated sum of squared loadings		
	Total	Variance %	Cumulative%	Total	Variance %	Cumulative%	Total	Variance %	Cumulative%
1	9.349	49.204	49.204	9.349	49.204	49.204	4.112	21.641	21.641
2	2.092	11.009	60.213	2.092	11.009	60.213	3.728	19.622	41.262
3	1.634	8.602	68.814	1.634	8.602	68.814	3.347	17.613	58.875
4	1.223	6.438	75.252	1.223	6.438	75.252	3.112	16.377	75.252
5	0.962	5.061	80.314						

This study conducted a factor analysis on external influence, self-cognition, psychological construction, and anxiety performance to extract common factors across dimensions. Factor analysis assumes correlations among variables. When a factor shows no significant correlation, it is difficult to identify the dominant common factors. Therefore, a preliminary correlation analysis is necessary. The validity analysis revealed that the average KMO value was 0.862 (greater than 0.6), and the *p*-value of the Bartlett’s test was approximately 0.000, which verified that there were statistically significant differences between the original correlation coefficient matrix and the principal component matrix. Consequently, factor analysis was performed on the original components via principal component analysis for dimensionality reduction.

#### 3.2.1 External influences and self-cognition: exploratory factor analysis

In the factor analysis, the rotation matrix of the scale was derived from five items each of external influence and student self-cognition. As shown in Table 5, this factor analysis extracted two principal components, and all the dimension factor loadings exceeded 0.5. This suggests that the questionnaire items exhibit good structural validity and lays a foundation for subsequent research.

**Table 5:** Exploratory factor analysis results of external influence and self-cognition.

Items	Rotated component matrix a	
	Factor 1	Factor 2
WJ1	0.717	
WJ2	0.694	
WJ3	0.677	
WJ4	0.730	
WJ5	0.751	
ZW1		0.712
ZW2		0.718
ZW3		0.796
ZW4		0.776
ZW5		0.804

#### 3.2.2 Exploratory factor analysis of psychological construction and employment anxiety

Factor analysis of the rotation component matrix of the observation scale (Table 6) indicates that the variables within each factor display loadings that are solely associated with one factor. For example, variable XL1 (insufficient preparation for social identity transition) shows a relatively higher loading on the first factor, whereas variable JV3 (a growing sense of urgency as graduation approaches) exerts a significant loading effect on the second factor.

**Table 6:** Exploratory factor analysis results of psychological construction and employment anxiety.

Items	Rotated component matrix a	
	Factor 1	Factor 2
XL1	0.870	
XL2	0.867	
XL3	0.763	
XL4	0.717	
XL5	0.601	
JV1		0.517
JV2		0.605
JV3		0.873
JV4		0.827
JV5		0.766

### 3.3 Confirmatory factor analysis (CFA)

The current study employed confirmatory factor analysis (CFA) to assess the structural validity of the four-dimensional model (comprising self-cognition, psychological construction, external influence, and anxiety performance). The key fit indices were as follows: χ^2^/df = 2.37 (≤3, regarded as acceptable); RMSEA = 0.076 (≤0.08, suggesting a favorable fit); CFI = 0.92; TLI = 0.91 (both >0.90, regarded as optimal).

The item ZW2, which exhibited a high cross-loading (self-cognition-psychological construction, cross-loading = 0.32), was eliminated, leading to a substantial enhancement in model fit (Δχ^2^ = 18.74, *p* < 0.001). The composite reliability (CR) values for each dimension spanned from 0.83 to 0.89 (all > 0.7), and the average variance extracted (AVE) ranged from 0.56 to 0.67 (all > 0.5), suggesting favorable reliability and validity of the scale. Table 7 indicates that the four-dimensional model demonstrates a good fit with the data, as all indicators meet psychometric criteria, validating the rationality of the scale structure. Factor loadings indicated that all items had loadings between 0.62 and 0.85 (*p* < 0.001) on their corresponding dimensions, further corroborating construct validity. These findings further confirm the structural stability of the four-dimensional model, providing a measurement basis for subsequent hypothesis testing.

**Table 7:** CFA factor loadings matrix (standardized loadings, *p* < 0.001).

Items	Self-cognition	Psychological construction	External influence	Anxiety performance
ZW1	0.78	-	-	-
ZW3	0.82	-	-	-
ZW5	0.75	-	-	-
XL2	-	0.79	-	-
XL4	-	0.85	-	-
WJ2	-	-	0.73	-
WJ5	-	-	0.68	-
JV3	-	-	-	0.81
JV4	-	-	-	0.76

### 3.4 Descriptive statistics of survey questionnaires

This questionnaire survey was distributed among public and private schools, with the target population being the graduating classes of 2023 and 2024. Among the valid responses, 50.62% were from public schools and 49.38% were from private schools. Additionally, 50.21% were from the class of 2023 graduates, and 49.79% were from the class of 2024 graduates. The relevant details are presented in Table 8.

**Table 8:** Statistical analysis of personal characteristics of Chinese language teaching department students in Taiwan, China (*N* = 241).

Dimension	Category	*N*	Proportion (%)
School	Public	122	50.62
	Private	119	49.38
Gender	Male	75	31.12
	Female	166	68.88
Graduation year	2023	121	50.21
	2024	120	49.79
Identity	General identity includes new residents	221	91.70
	Aborigines	20	8.30
Number of children in one family	Only child	71	29.46
	2 children	132	54.77
	> 3 children	38	15.77
Monthly household income (NT$)^a^	<30,000	20	8.3
	30,000–80,000	57	23.65
	80,000–150,000	112	46.47
	>150,000	52	21.58
Foreign language aptitude (multiple choice)	No	34	14.10
	TOEIC 550 + or other equivalent grades	196	81.32
	Japanese N2 or above, or equivalent proficiency	67	27.80
	Korean language proficiency test level 4 or above, or equivalent qualification	8	3.31
	Others	6	2.48
Specialty (multiple choice)	No	58	24.06
	Sports	96	39.83
	Language and arts	74	30.70
	Traditional culture	46	19.08
	Others	1	0.41
Join a student union or club	Yes	167	69.29
	No	74	30.71
Internship experience	Participate in matched internship	136	56.43
	Participate in non-matched internships	44	18.26
	No internship experience	61	25.31
Planned future employment area	Northern region	119	49.38
	Midland	60	24.90
	Southern region	40	16.60
	Others	22	9.13
Target employment choice	Yes	139	57.68
	No	75	31.12
	Not decide	27	11.20
Expected monthly salary range (NT$)	<30,000	76	31.54
	30,000–80,000	117	48.55
	80,000–150,000	42	17.43
	>150,000	6	2.49
Occupational planning	Yes	154	63.90
	No	87	36.10
The critical time to start cognitive career planning	Before entering university	81	33.61
	During my university years	134	55.60
	Upon graduation	26	10.79
Ways to recognize the importance of career planning (multiple choice)	Through information provided by elders and friends	201	83.40
	Social news and social media, etc.	34	14.10
	the case in school curriculum	84	34.85
Understanding of employment trends and policies for desired careers	No	80	33.20
	I have some knowledge, but the information is limited	98	40.66
	I have learned and acquired a wealth of information.	63	26.14

a The data is based on salary information from 104 Job Bank, a job-seeking platform in Taiwan, China.

#### 3.4.1 Internal conditions of students

Table 8 indicates that among the students from the four surveyed schools, 91.70% are general new residents, while 8.3% are indigenous residents. The gender distribution exhibits a notable disparity, with females accounting for 68.88% and males 31.12%. The family structure sample is classified into two dimensions: family members and monthly household income. In the aspect of family composition, 54.77% of the students have two siblings, 29.46% are only children, and 15.77% have three or more siblings. Concerning monthly income, 8.3% of households have a monthly income of NT$30,000 or below, 23.65% have an income ranging from NT$30,000 to NT$80,000, 46.47% have an income between NT$80,000 and NT$150,000, and 21.58% belong to high-income households with an income of NT$150,000 or above. This suggests that the majority of students majoring in Chinese Language Education in Taiwan, China, possess relatively stable family conditions.

#### 3.4.2 Student choice

Survey data on university life reveal that 69.29% of students have participated in student unions or clubs, while 30.71% have not been actively involved in these organizations. Among the surveyed undergraduates, 74.69% have completed pre-employment internships. Specifically, 56.43% have interned in their respective fields of study, and 25.31% have no prior internship experience.

The findings from the questionnaire indicate that Chinese language majors in Taiwan, China, predominantly exhibit a strong inclination towards seeking employment in the northern regions. Specifically, 49.38% of the respondents intend to pursue their careers in this area, citing the region’s favorable employment outlook and ample opportunities for professional advancement as the primary reasons for their preference. Respondents targeting central Taiwan account for 24.9%, followed by those aiming for the southern regions at 16.6%, while 9.13% choose to seek employment overseas.

Regarding career trajectories, 57.68% of the respondents plan to engage in field-specific occupations upon graduation, 11.2% are indecisive, and 31.12% have firmly determined not to pursue related professions.

#### 3.4.3 Individual abilities of students

The survey reveals that English remains the predominant primary foreign language among students, accounting for 81.32%. Japanese is chosen as the primary foreign language by 27.80% of students; however, only a small proportion of students are proficient in both languages. Meanwhile, 14.10% of students lack any foreign-language proficiency. In the Chinese Language Teaching Department, students are encouraged to develop specialized competencies. Notably, nearly equal proportions (39.83 and 30.70%) demonstrate strengths in sports and language arts respectively, while 24.06% of students state that they possess no particular aptitudes.

31.54% of students expect a monthly salary of NT$30,000 or less. Nearly half (48.55%) anticipate earning between NT$30,000 and NT$80,000 per month, whereas 17.43% aspire to earn between NT$80,000 and NT$150,000. Only 2.49% of students envision a monthly salary of NT$150,000 or more.

#### 3.4.4 Future planning for students

Table 8 reveals that 63.9% of students have career plans, with nearly half formulating professional goals and seeking employment in specific fields during their academic studies. Specifically, 33.61% initiated career planning before enrolling in college, and 55.6% developed career strategies through specialized curricula. Career awareness was primarily acquired through the following channels: 83.40% from family and friends, 34.85% via school case analyses, and 14.10% through digital media platforms. Regarding industry knowledge, 66.8% were aware of employment trends and policies, with 26.14% possessing in-depth industry-specific expertise and 40.66% having a general understanding. Notably, 36.1% had no career planning, 33.2% were unaware of employment policies during college, and 10.9% only began career planning close to graduation.

### 3.5 Descriptive statistics of employment anxiety variables among participants

A descriptive statistical analysis was conducted on the five-item scale, including the maximum, minimum, mean, and standard deviation, as shown in Tables 9. The scale employs a 5-point scale spanning from strongly agree to strongly disagree. The data indicate that students majoring in Chinese language teaching in Taiwan, China, face relatively high levels of employment anxiety. The average scores of all items reveal that the university students from the 2023 and 2024 cohorts experienced campus life during the pandemic. They generally considered four variables as factors contributing to employment anxiety: WJ2 (the impact of the pandemic on employment influencing them, M = 2.77), WJ5 (internships not promoting job prospects, M = 2.77), XL5 (difficulty in obtaining satisfactory jobs with a bachelor’s degree, M = 2.77), and JV4 (interview anxiety despite thorough preparation, M = 2.77). Collectively, these four variables emerged as the most significant factors contributing to employment anxiety among university students.

**Table 9:** Descriptive statistics of employment anxiety variables among Chinese language teaching majors in Taiwan, China (*N* = 241).

Descriptive variables	N	Min	Max	Mean	SD
ZW1	241	1	5	2.81	1.479
ZW2	241	1	5	3.06	1.221
ZW3	241	1	5	2.93	1.362
ZW4	241	1	5	3.05	1.333
ZW5	241	1	5	3.23	1.430
WJ1	241	1	5	3.02	1.199
WJ2	241	1	5	2.77	1.262
WJ3	241	1	5	3.03	1.346
WJ4	241	1	5	3.13	1.302
WJ5	241	1	5	2.77	1.309
XL1	241	1	5	3.01	1.199
XL2	241	1	5	2.84	1.292
XL3	241	1	5	3.03	1.341
XL4	241	1	5	3.12	1.300
XL5	241	1	5	2.77	1.318
JV1	241	1	5	2.99	1.340
JV2	241	1	5	2.98	1.478
JV3	241	1	5	3.03	1.206
JV4	241	1	5	2.77	1.267
JV5	241	1	5	3.04	1.346

## 4 Discussion

### 4.1 Differences in employment anxiety among students in the Chinese language teaching department

#### 4.1.1 Differences among different types of schools

Table 10 presents the mean and standard deviation of 20 five-point scale items among the surveyed students from four public and private schools in Taiwan, China. The standard deviation of the four dimensions for graduates of public universities ranges from 1.266 to 1.415, while that for graduates of private universities ranges from 1.125 to 1.492. Concerning the overall anxiety levels across public and private schools, only the self-cognition dimension demonstrated statistically significant differences (*p* < 0.01), and the *P*-values of the other three dimensions all exceeded 0.05. This indicates that there are no significant discrepancies in employment anxiety between public and private school students in terms of external influence, psychological construction, and anxiety manifestation. Notably, in the self-cognition dimension where significant differences were identified, private school students exhibited lower anxiety levels compared to the average, whereas public school students had an average score above 3.0. This suggests that public school students display more prominent anxiety in the self-cognition dimension than private school students.

**Table 10:** Analysis results of anxiety differences among students in different types of schools.

Dimension	Variable	*N*	Mean	SD	T
Self-cognition	Private	119	3.101	1.492	3.076***
	Public	122	2.525	1.415	
	Total	241	2.809	1.479	
External influence	Private	119	3.076	1.129	0.701
	Public	122	2.967	1.266	
	Total	241	3.021	1.199	
Psychological construction	Private	119	3.067	1.125	0.7
	Public	122	2.959	1.269	
	Total	241	3.012	1.199	
Anxiety performance	Private	119	3.042	1.298	0.622
	Public	122	2.934	1.383	
	Total	241	2.988	1.34	

*** represent significance levels of 1%.

The data in Table 10, obtained from the LSD test, indicated that students in public schools demonstrated significantly higher anxiety levels in the self-perception dimension than those in private schools (mean score of public school students = 2.525, mean score of private school students = 3.101, mean difference = 0.576, standard error = 0.187, *p* < 0.01). Specifically, the scores of public school students in self-perception were significantly lower than the mid-point of the scale (3.0), whereas the scores of private school students were approaching the mid-point. This implies that public school students had more prominent negative evaluations of their own capabilities (e.g., teaching skills, career competitiveness).

In dimensions such as external influences, psychological development, and anxiety manifestations, no statistically significant differences were detected between public and private school students (*p* > 0.05). This suggests that the type of school primarily impacts students’ self-perception anxiety, with a limited influence on other anxiety dimensions. These findings suggest that public schools should reinforce the cultivation of students’ self-efficacy.

#### 4.1.2 Gender differences

Table 11 presents the mean and standard deviation of 20 items from the five-dimensional scale among the surveyed students. The independent samples T-test revealed gender differences in anxiety levels. Across all four dimensions, males exhibited standard deviations ranging from 1.166 to 1.507, whereas females had standard deviations ranging from 1.213 to 1.463. Notably, females consistently achieved lower scores than males in all four dimensions, suggesting higher anxiety levels.

**Table 11:** Gender differences in anxiety.

Dimension	Gender	*N*	Mean	SD	T
Self-cognition	Male	75	3	1.507	1.349
	Female	166	2.723	1.463	
	Total	241	2.809	1.479	
External influence	Male	75	3.133	1.166	0.98
	Female	166	2.97	1.213	
	Total	241	3.021	1.199	
Psychological construction	Male	75	3.133	1.166	1.052**
	Female	166	2.958	1.213	
	Total	241	3.012	1.199	
Anxiety performance	Male	75	3.053	1.218	0.511
	Female	166	2.958	1.394	
	Total	241	2.988		

** represent significance levels of 5%.

Previous relevant studies have shown that, generally, female graduates experience a higher level of employment-related anxiety than male graduates (e.g., Li, 2022; Zhang et al., 2012; Shu and Tang, 2008). The findings of this study are consistent with their conclusions.

#### 4.1.3 Differences in family circumstances

In the context of Taiwan, China, the majority of households have multiple children. Table 12 conducts a one-way analysis of variance (F-test) to compare the employment anxiety levels among different family sizes. It displays the mean scores and standard deviations of the surveyed students across 20 five-point scale items. Through Sphericity test analysis, four distinct dimensions were recognized.

**Table 12:** Differences in employment anxiety among students based on family sibling count.

Dimension	Variable	*N*	Mean	SD	F
Self-cognition	1 child	71	2.972	1.512	4.072**
	2 children	132	2.583	1.42	
	>3 children	38	3.289	1.505	
	Total	241	2.809	1.479	
External influence	1 child	71	2.972	1.195	0.16
	2 children	132	3.061	1.209	
	>3 children	38	2.974	1.197	
	Total	241	3.021	1.199	
Psychological construction	1 child	71	2.944	1.206	0.193
	2 children	132	3.053	1.213	
	>3 children	38	3	1.162	
	Total	241	3.012	1.199	
Anxiety performance	1 child	71	2.972	1.394	0.497
	2 children	132	2.939	1.324	
	>3 children	38	3.184	1.312	
	Total	241	2.988	1.34	

** represent significance levels of 5%.

In the comparison of employment anxiety based on family size, the standard deviation of only children ranged from 1.195 to 1.612; that of two-child families ranged from 1.209 to 1.42; and that of families with three or more siblings ranged from 1.162 to 1.505. Students from two-child families showed higher average scores in the dimensions of external influence and psychological construction, indicating lower anxiety levels compared to only children and those from families with multiple siblings. In terms of anxiety manifestation, there were negligible differences between only children and students from two-child families, while students from families with three or more siblings presented lower anxiety levels.

Table 12 demonstrates notable disparities between only children and children with siblings in self-cognition (*p* < 0.05), while for the other three dimensions, *p* > 0.1. Students with three or more siblings exhibit a mean self-cognition score exceeding 3.0, signifying a more comprehensive self-perception and lower levels of employment-related anxiety. Those from two-child families obtain a score of 2.583, which is below the overall average, implying stronger employment anxiety in comparison to only children.

Post-hoc multiple comparisons (LSD test) indicated significant disparities in self-cognition between the group of two children and the group of more than three children (*p* < 0.05), as well as between the only-child group and the group of more than three children (*p* < 0.05). No significant difference was detected between the only-child group and the group of two children (*p* > 0.05).

Tuition fees and living expenses constitute significant financial burdens for families funding higher education. Table 12 employs a one-way ANOVA F-test to analyze and compare the employment anxiety levels among students from families with different monthly incomes, yielding the following results: Among the four dimensions of employment anxiety, the self-cognition dimension shows the most notable difference (*p* < 0.10), with a mean difference of 0.432. Students from families with monthly incomes between NT$30,000 and NT$80,000 consistently report higher mean scores than the overall average. Conversely, students from families with higher or lower incomes experience more severe employment anxiety arising from self-cognition problems.

As shown in Table 13, the standard deviations for distinct family income categories are as follows: below NT$30,000 (standard deviation ranging from 1.129 to 1.418), NT$30,000-NT$80,000 (standard deviation ranging from 1.083 to 1.337), NT$80,000-NT$150,000 (standard deviation ranging from 1.209 to 1.536), and above NT$150,000 (standard deviation ranging from 1.302 to 1.512). Students from families with monthly incomes between NT$80,000 and NT$150,000 exhibit lower mean scores in the dimensions of self-cognition, external influence, and psychological construction compared to the overall average. This indicates that students within this income bracket are more susceptible to internal and external influences when making career decisions. Students from families with monthly incomes below NT$30,000, between NT$30,000 and NT$80,000, or above NT$150,000 present lower mean anxiety scores compared to the overall average. Their scores tend to approach 3.0 as income increases, whereas other students are more likely to exhibit anxiety-related behaviors during career selection.

**Table 13:** Differences in employment anxiety among students based on household monthly income (NT$).

Dimension	Variable	*N*	Mean	SD	F
Self-cognition	<30,000	20	2.7	1.418	1.283*
	30,000–80,000	57	3.123	1.337	
	80,000–150,000	112	2.768	1.536	
	>150,000	52	2.596	1.512	
	Total	241	2.809	1.479	
External influence	<30,000	20	3.3	1.129	0.695
	30,000–80,000	57	3.07	1.083	
	80,000–150,000	112	2.92	1.209	
	>150,000	52	3.077	1.326	
	Total	241	3.021	1.199	
Psychological construction	<30,000	20	3.3	1.129	0.805
	30,000–80,000	57	3.053	1.093	
	80,000–150,000	112	2.902	1.215	
	>150,000	52	3.096	1.302	
	Total	241	3.012	1.199	
Anxiety performance	<30,000	20	2.9	1.334	0.345
	30,000–80,000	57	2.86	1.246	
	80,000–150,000	112	3.071	1.347	
	>150,000	52	2.981	1.448	
	Total	241	2.988	1.34	

* represent significance levels of 10%.

Post-hoc multiple comparisons (LSD test) revealed significant disparities in the self-cognition dimension. Specifically, students from families with monthly incomes ranging from NT$30,000 to NT$80,000 exhibited significantly higher levels of anxiety compared to those from families with monthly incomes between NT$80,000 and NT$150,000 (*p* < 0.05) and those from families with monthly incomes exceeding NT$150,000 (*p* < 0.01). Conversely, no significant difference was detected between the group with monthly incomes of NT$80,000-NT$150,000 and the group with monthly incomes above NT$150,000 (*p* > 0.05).

#### 4.1.4 Differences in learning ability

Proficiency in a foreign language is regarded as a fundamental competency for graduates of the Chinese Language Teaching Department. Competence in a foreign language not only promotes teaching effectiveness but also improves students’ understanding. This multiple-choice question utilizes variable coding: 1 represents no foreign-language proficiency, 2 indicates a TOEIC score of 550 or higher, 3 denotes a Japanese N2 or a higher level, 4 signifies a Korean CET-4 or a higher level, and 5 represents other foreign-language proficiencies, presented in the order of the questionnaire.

As shown in Table 14, the four-dimensional Sphericity test demonstrates that there is no significant disparity in students’ foreign-language proficiency with respect to personal employment anxiety (*p* > 0.10).

**Table 14:** Differences in employment anxiety among students based on foreign language proficiency.

Dimension	Variable coding	*N*	Mean	SD	F
Self-cognition	1	34	3.235	1.415	1.552
	2	156	2.714	1.476	
	3	32	2.625	1.519	
	4	6	2.333	1.366	
	5	5	3.6	1.14	
	2, 5	1	5	0.000	
	3, 2	5	3.6	1.517	
	3, 4	1	5	0.000	
	4, 2	1	2	0.000	
	Total	241	2.809	1.479	
External influence	1	34	2.765	0.855	1.003
	2	156	2.968	1.212	
	3	32	3.219	1.475	
	4	6	3.5	1.049	
	5	5	3.2	1.095	
	2, 5	1	5	0.000	
	3, 2	5	3.6	0.894	
	3, 4	1	3	0.000	
	4, 2	1	3	0.000	
	Total	241	3.021	1.199	
Psychological construction	1	34	2.765	0.855	1.095
	2	156	2.961	1.209	
	3	32	3.219	1.475	
	4	6	3.5	1.049	
	5	5	3.2	1.095	
	2, 5	1	5	0.000	
	3, 2	5	3.6	0.894	
	3, 4	1	3	0.000	
	4, 2	1	2	0.000	
	Total	241	3.012	1.199	
Anxiety performance	1	34	2.647	1.012	1.092
	2	156	2.974	1.395	
	3	32	3.062	1.435	
	4	6	3.333	1.211	
	5	5	2.8	1.304	
	2, 5	1	5	0.000	
	3, 2	5	3.6	0.894	
	3, 4	1	4	0.000	
	4, 2	1	4	0.000	
	Total	241	2.988	1.34	

Post-hoc multiple comparisons (LSD test) further suggest that there are no significant differences in employment anxiety among all foreign-language proficiency groups (all *p* > 0.05). Nevertheless, students with TOEIC scores of 550 or higher (Group 2) presented the lowest mean scores in self-cognition (2.64) and anxiety performance (2.81), whereas those without foreign-language ability (Group 1) displayed the highest mean scores in external influence (3.12) and psychological construction (3.08).

Among the students’ responses, the group with English as their only first foreign language was the largest. The standard deviation of this group ranged from 1.209 to 1.476. Their average scores across all four dimensions were below the overall mean, indicating more pronounced employment anxiety. Students who perceived themselves as deficient in foreign-language skills had a standard deviation between 0.855 and 1.415. Except for the self-cognition dimension, their average scores in the other three dimensions were lower than the overall average, making them more susceptible to anxiety during the job-hunting process due to external demands and competition.

Overall, students proficient in both English and other minor languages manifested low levels of anxiety. Students proficient in both English and Japanese presented notably higher average scores across all four dimensions in comparison to the overall average, suggesting minimal anxiety.

In the context of employment for Chinese language teaching majors, the acquisition of specialized skills can improve teaching effectiveness and enable students to appreciate the aesthetic essence of Chinese culture. This multiple-choice question utilizes variable coding from 1 to 5, corresponding, respectively, to the lack of special skills, sports-related skills, language and arts skills, traditional culture skills, and other skills, arranged in the sequence of the questionnaire.

Table 15 employs one-way ANOVA and the F-test to analyze the disparities in employment anxiety among students in relation to their individual skills. The findings reveal significant differences across all three dimensions, with the exception of self-cognition (*p >* 0.05). Students possessing traditional culture skills exhibit standard deviations spanning from 1.358 to 1.54, and the means of all four dimensions are lower than the overall average, indicating relatively elevated levels of employment anxiety. Those who perceive themselves as lacking special skills present standard deviations between 0.823 and 1.33, and the means of all three dimensions, excluding self-cognition, are below the overall average. This reflects self-assurance in job-seeking, yet also reveals anxiety stemming from external requirements and skill comparisons. Overall, students with both sports and language arts skills have the highest standard deviation (ranging from 0.947 to 1.472), followed by those with both language arts and traditional culture skills (ranging from 1.118 to 1.5). Although these students manifest self-confidence and comprehensive self-awareness, their employment anxiety levels remain relatively high.

**Table 15:** Differences in employment anxiety among students with special skills.

Dimension	Variable coding	*N*	Mean	SD	F
Self-cognition	1	58	3.138	1.33	1.282
	2	76	2.48	1.398	
	3	48	2.771	1.64	
	4	30	2.8	1.54	
	2, 3	13	3	1.472	
	2, 4	3	3.667	2.309	
	3, 4	9	2.667	1.5	
	2, 3, 4	3	3.667	1.528	
	2, 3, 4, 5	1	5	0.000	
	Total	241	2.809	1.479	
External influence	1	58	2.914	0.823	2.347**
	2	76	3.147	1.291	
	3	48	3.229	1.259	
	4	30	2.467	1.383	
	2, 3	13	3.308	0.947	
	2, 4	3	4.333	1.155	
	3, 4	9	2.333	1.118	
	2, 3, 4	3	2.667	0.577	
	2, 3, 4, 5	1	5	0.000	
	Total	241	3.021	1.199	
Psychological construction	1	58	2.897	0.831	2.341**
	2	76	3.133	1.298	
	3	48	3.229	1.259	
	4	30	2.5	1.358	
	2, 3	13	3.308	0.947	
	2, 4	3	4.333	1.155	
	3, 4	9	2.333	1.118	
	2, 3, 4	3	2.333	0.577	
	2, 3, 4, 5	1	5	0.000	
	Total	241	3.012	1.199	
Anxiety performance	1	58	2.724	1.225	2.237**
	2	76	2.84	1.366	
	3	48	3.417	1.35	
	4	30	2.967	1.402	
	2, 3	13	3.538	1.05	
	2, 4	3	4.333	1.155	
	3, 4	9	2.444	1.236	
	2, 3, 4	3	3	1	
	2, 3, 4, 5	1	5	0.000	
	Total	241	2.988	1.34	

** represent significance levels of 5%.

Post-hoc multiple comparisons (LSD test) indicated significant disparities in external influence. Specifically, students possessing traditional culture skills (Group 4) exhibited higher levels of anxiety compared to those with language arts skills (Group 3) (*p* < 0.05) and those with sports-related skills (Group 2) (*p* < 0.05). In terms of psychological construction, students without special skills (Group 1) demonstrated higher anxiety than those with language arts skills (Group 3) (*p* < 0.05). No significant differences were detected in anxiety performance among the skill groups (all *p* > 0.05).

#### 4.1.5 Differences in student activity experience

Table 16 presents the results of an independent samples T-test examining the impact of student club participation on employment anxiety. All the outcomes suggest that *p* > 0.10, indicating no significant disparities. Among the surveyed students, the majority participated in campus activities (SD: 1.253–1.516). The mean value of the self-cognition dimension of these students was lower than the overall average, which suggests strong competitiveness but low self-confidence. The minority of non-participants (SD: 1.072–1.394) showed lower means in the dimensions of external influence, psychological construction, and anxiety performance compared to the overall average. Notably, the mean of their anxiety performance dimension was 0.015 lower than the overall average, signifying the most substantial difference. This implies that although these students did not face intense competition on campus, they developed anxiety-related psychological and behavioral responses due to external influences during the job-seeking process. Overall, students without campus activity participation demonstrated relatively mild anxiety levels.

**Table 16:** Employment anxiety among students with different activity experiences.

Dimension	Variable	N	Mean	SD	T
Self-cognition	No	75	2.947	1.394	0.97
	Yes	166	2.747	1.516	
	Total	241	2.809	1.479	
External influence	No	75	3.013	1.072	−0.064
	Yes	166	3.024	1.255	
	Total	241	3.021	1.199	
Psychological construction	No	75	3	1.078	−0.108
	Yes	166	3.018	1.253	
	Total	241	3.012	1.199	
Anxiety performance	No	75	2.973	1.208	−0.11
	Yes	166	2.994	1.399	
	Total	241	2.988	1.34	

Post-hoc multiple comparisons (LSD test) revealed that within the anxiety performance dimension, non-matched internship students exhibited significantly higher levels of anxiety compared to matched internship students (*p* < 0.01) and non-internship students (*p* < 0.05). Moreover, no significant difference was detected between matched internship students and non-internship students (*p* > 0.05).

#### 4.1.6 Differences in internship work experience

Graduation internships represent non-compulsory requirements, and a substantial proportion of students regard them as a method to improve their professional competencies. Not all students choose to participate in internships. Table 17 employs a one-way ANOVA F-test to analyze and compare the differences in employment anxiety levels among students with various internship experiences.

**Table 17:** Employment anxiety differences in student-related internship experiences.

Dimension	Variable	*N*	Mean	SD	F
Self-cognition	Participate in non-matched internships	44	2.977	1.389	2.288
	Participate in matched internships	137	2.635	1.46	
	No internship	60	3.083	1.555	
	Total	241	2.809	1.479	
External influence	Participate in non-matched internships	44	3	1.258	0.257
	Participate in matched internships	137	2.985	1.207	
	No internship	60	3.117	1.151	
	Total	241	3.021	1.199	
Psychological construction	Participate in non-matched internships	44	3.023	1.229	0.162
	Participate in matched internships	137	2.978	1.209	
	No internship	60	3.083	1.169	
	Total	241	3.012	1.199	
Anxiety performance	Participate in non-matched internships	44	2.432	1.437	4.778***
	Participate in matched internships	137	3.117	1.289	
	No internship	60	3.1	1.298	
	Total	241	2.988	1.34	

*** represent significance levels of 1%.

An analysis of the four dimensions of employment anxiety across diverse internship experiences indicates that for students who have participated in relevant internships, the standard deviation falls within the range of 1.207 to 1.46. For those involved in unrelated internships, the standard deviation lies between 1.229 and 1.437. In the case of non-internship students, the standard deviation ranges from 1.298 to 1.555, which is the widest among the three groups. The most significant difference is detected in the anxiety performance dimension (*p* < 0.01), whereas the other three dimensions do not display notable disparities.

In the dimension of anxiety performance, the average scores of students with non-relevant internships are lower than the overall average, which implies a relatively higher level of employment-related anxiety. In the dimensions of self-cognition, external influence, and psychological construction, the average scores of students with relevant internships are lower compared to the overall average, indicating that they experience substantial but unexpressed anxiety.

Post-hoc multiple comparisons (LSD test) indicated significant disparities in external influence. Specifically, students from the Northern region exhibited lower levels of anxiety compared to overseas students (*p* < 0.05) and students from the Southern region (*p* < 0.1). In terms of psychological construction, students from the Northern region had lower anxiety levels than overseas students (*p* < 0.05).

#### 4.1.7 Differences in pre-employment preparation

Prior to their entry into the labor market, all students harbor expectations regarding their future living environments. This section aggregates students’ individual plans for potential employment regions. Table 18 employs a one-way analysis of variance (ANOVA) to investigate whether students’ planned employment locations intensify employment anxiety and the associated disparities.

**Table 18:** Employment anxiety by expected employment region.

Dimension	Variable	*N*	Mean	SD	F
Self-cognition	Northern region	119	2.664	1.48	1.38
	midland	60	3.117	1.485	
	Southern region	40	2.875	1.343	
	Others	22	2.636	1.649	
	Total	241	2.809	1.479	
External influence	Northern region	119	3.16	1.249	2.515*
	midland	60	3.067	1.056	
	Southern region	40	2.85	1.075	
	Others	22	2.455	1.371	
	Total	241	3.021	1.199	
Psychological construction	Northern region	119	3.151	1.253	2.427*
	midland	60	3.05	1.064	
	Southern region	40	2.85	1.051	
	Others	22	2.455	1.371	
	Total	241	3.012	1.199	
Anxiety performance	Northern region	119	3.05	1.364	1.764
	midland	60	3.133	1.255	
	Southern region	40	2.9	1.297	
	Others	22	2.409	1.436	
	Total	241	2.988	1.34	

* represent significance levels of 10%.

The results reveal that the highest number of students expect to work in northern regions, with standard deviations ranging from 1.249 to 1.48. In this group, students have mean values in the self-cognition dimension below 3.0, which is lower than the overall average, suggesting self-uncertainty and a greater predisposition to anxiety. Among the four dimensions tested for Sphericity, significant differences are detected in external influence and psychological construction (*p <* 0.1). Students who choose southern or overseas locations have lower mean values in the external influence, psychological construction, and anxiety performance dimensions compared to the overall average, indicating a higher degree of anxiety than those expecting to work in the north.

Overall, the smallest number of students aspire to work overseas, yet their four-dimensional values remain below the average, making them the most anxious during the employment process. In contrast, students planning to work in the Taichung region demonstrate better performance across all dimensions, with all four mean values surpassing the overall average, indicating the least regional impact on employment anxiety.

Career planning holds paramount importance for students making the transition into the labor market. A meticulously designed career plan can establish a robust foundation for professional development and enable individuals to actualize their career potential. This study utilized an independent samples T-test to investigate the differences in employment anxiety between students with and without career planning.

As shown in Table 19, within the four dimensions of employment anxiety, only the self-cognition dimension exhibited significant differences (*p <* 0.05), while the other three dimensions did not display remarkable variations. In this survey, the number of students with career planning was approximately twice that of students without. The standard deviation of students with career planning fell within the range of 1.16 to 1.495. Except for anxiety manifestation, the mean scores of the other three dimensions were lower than the overall average, which implies that despite having career plans, these students still suffered from anxiety due to a lack of confidence in the job market and their own competencies. Conversely, the standard deviation of students without career planning ranged from 1.256 to 1.459. Except for anxiety manifestation, the other three dimensions presented lower anxiety levels compared to the overall average, indicating that such students primarily demonstrated anxiety-related characteristics when faced with employment difficulties.

**Table 19:** Difference analysis of employment anxiety between students with and without pre-employment planning.

Dimension	Variable	N	Mean	SD	T
Self-cognition	Yes	154	2.805	1.495	3.055**
	No	87	2.816	1.459	
	Total	241	2.809	1.479	
External influence	Yes	154	2.961	1.16	−1.029
	No	87	3.126	1.265	
	Total	241	3.021	1.199	
Psychological construction	Yes	154	2.948	1.165	−1.11
	No	87	3.126	1.256	
	Total	241	3.012	1.199	
Anxiety performance	Yes	154	3.013	1.338	0.391
	No	87	2.943	1.35	
	Total	241	2.988	1.34	

** represent significance levels of 5%.

For newly graduated individuals aiming to devise comprehensive, practical, and achievable career plans, understanding the significance of such plans is of paramount importance, as planning strategies vary across different stages. Table 20 employs one-way ANOVA F-tests to examine the temporal variations in employment anxiety levels stemming from students’ perceptions of the importance of career planning. The results suggest that the mean values of all four dimensions (*p* > 0.10) do not display significant disparities.

**Table 20:** Analysis of employment anxiety caused by temporal differences in students’ recognition of career planning importance.

Dimension	Variable	*N*	Mean	SD	F
Self-cognition	Before entering university	81	2.679	1.54	0.486
	Upon graduation	26	2.923	1.573	
	During university years	134	2.866	1.429	
	Total	241	2.809	1.479	
External influence	Before entering university	81	2.951	1.322	0.684
	Upon graduation	26	2.846	1.377	
	During university years	134	3.097	1.082	
	Total	241	3.021	1.199	
Psychological construction	Before entering university	81	2.951	1.322	0.763
	Upon graduation	26	2.808	1.386	
	During university years	134	3.09	1.079	
	Total	241	3.012	1.199	
Anxiety performance	Before entering university	81	2.926	1.412	0.131
	Upon graduation	26	3	1.414	
	During university years	134	3.022	1.289	
	Total	241	2.988	1.34	

When examining the influence of different stages of career-planning awareness on employment anxiety across four dimensions, students who developed initial awareness before entering college exhibited standard deviations within the range of 1.322 to 1.54. Students who developed awareness during their college tenure had standard deviations between 1.079 and 1.429. In contrast, students who recognized the significance of career planning only near graduation had standard deviations ranging from 1.377 to 1.573. The maximum standard deviation was observed among students who became aware of its importance during their college years.

Students who recognized the significance of career planning prior to entering college presented lower mean values across all four dimensions when compared to the overall average. This indicates that individuals with early-stage planning manifested stronger competitive awareness and a heightened perception of employment competition. In contrast, students who initiated career planning close to graduation displayed lower standard deviations in the dimensions of external influence and psychological construction. This implies that these students lacked adequate psychological preparation and were more susceptible to environmental impacts.

#### 4.1.8 Summary

The survey results suggest that the employment anxiety among graduates of Chinese Language Teaching programs can be ascribed to a variety of intricate and multi-dimensional factors. External immutable factors have a significant impact on students’ self-perception, resulting in remarkable disparities in career selections. For instance, in the aspect of psychological constitution, female students display a higher level of anxiety than male students, and students from public schools manifest more prominent self-perception anxiety. Although external variable factors generally have a relatively limited influence on students’ anxiety levels, their employment decisions are influenced by changes in the external environment.

The survey indicates that the majority of students primarily utilize English as their first foreign language. Individuals seeking employment exclusively in English-related domains exhibit the highest levels of anxiety, whereas students proficient in both English and other less frequently used languages demonstrate relatively lower anxiety. Students with strong proficiency in traditional culture and linguistic arts possess comprehensive self-awareness yet still encounter substantial anxiety.

During the graduation phase, employment success is dependent on both personal capabilities and family support. Students from families with three or more siblings have a well-developed self-perception and lower employment-related anxiety, while those from families with two siblings display more pronounced anxiety. Students from families with a monthly income ranging from NT$80,000 to NT$150,000 are more susceptible to internal and external influences during the career-selection process.

There is no significant correlation between employment anxiety and active engagement in campus activities. Students with non-major internships experience the most severe anxiety, while those with major-related internships generally exhibit evident anxiety without overt signs.

Research findings indicate that students with pre-enrollment plans possess a more pronounced sense of competition, encounter higher levels of employment pressure, and undertake more systematic job-seeking preparations. The proportion of students with pre-employment plans is roughly double that of those without such plans. Although students with career plans exhibit marginally superior social adaptability, those lacking pre-employment planning tend to manifest more conspicuous anxiety during the job-hunting process. Owing to the unique characteristics of the Chinese Language Teaching Department, students with overseas development plans report the highest degrees of employment anxiety, and those desiring to work in the southern region display significantly greater anxiety compared to those targeting the northern region.

This investigation indicates that within an academic context characterized by involution, prospective teachers, subsequent to securing their teaching posts, ought to acquire the skills to alleviate stress, regulate their psychological states, and promote their professional growth. This will render teaching more dynamic and attractive, thus inspiring students’ enthusiasm for learning Chinese.

For variables that exhibited statistical significance in the analysis of variance (e.g., monthly household income and internship experience), pairwise comparisons among groups were conducted using the LSD test. As shown in Table 21, students in the “30,000–80,000” monthly household income group (M = 3.123) demonstrated significantly higher anxiety levels in the self-cognition dimension compared to the “80,000–150,000” group (M = 2.768) and the “150,000 + annually” group (M = 2.596). This suggests that students from middle-income families possess stronger self-doubt regarding their capabilities. The data in Table 22 reveal that the non-matched internship group (M = 2.432) presented significantly lower anxiety performance than the matched internship group (M = 3.117) and the no-internship group (M = 3.100). This is likely due to the lower career expectations among non-matched interns, resulting in milder anxiety.

**Table 21:** Impact of household monthly income on self-cognition (*F* = 1.283*, *p* < 0.10).

Control group (NT$)	Mean	SD	*P*
30,000 ~ 80,000 vs. 80,000 ~ 150,000	0.355	0.182	0.049*
30,000 ~ 80,000 vs. > 150,000	0.527	0.201	0.009**
80,000 ~ 150,000 vs. > 150,000	−0.172	0.179	0.338

**, and * represent significance levels of 5 and 10% respectively.

**Table 22:** Impact of internship experience on anxiety performance (*F* = 4.778***, *p* < 0.01).

Control group	Mean	SD	*P*
Non-aligned internship vs. Aligned internship	−0.685	0.231	0.003**
Non-matched internships vs. No internships	−0.668	0.254	0.009**
Corresponding internship vs. No internship	0.017	0.198	0.931

** represent significance levels of 5%.

Using employment anxiety scores as the dependent variable and self-cognition, external influence, and psychological construction as independent variables, a multiple linear regression analysis was conducted after controlling for demographic variables such as gender and school type. The results of the multicollinearity test indicated that the tolerance values ranged from 0.632 to 0.875 and the VIF values were between 1.143 and 1.582 (see Table 23), suggesting no significant multicollinearity problems (VIF < 10). The data in Table 23 shows that self-cognition has the most significant impact on employment anxiety (*β* = 0.382, *p* < 0.001), which implies that students’ negative self-appraisals, such as “inadequate teaching skills” or “feeling unable to stand out”, are the core sources of anxiety. Psychological construction follows closely (β = 0.251, *p* < 0.01), and psychological states like “lack of readiness for identity transition” and “unmet salary expectations” significantly exacerbate anxiety. External influence has relatively weaker effects (β = 0.179, *p* < 0.05), and factors such as insufficient career guidance and the impacts of the pandemic have a limited influence.

**Table 23:** Multiple linear regression of variables.

Predictive variable	Non-standardized coefficient	SD	Standardized coefficient	t	*P*	Effect strength ranking	Tolerance	VIF
Self-cognition	0.426	0.078	0.382	5.462	0.000***	1 (strongest)	0.782	1.279
Psychological construction	0.289	0.01	0.251	3.567	0.001**	2	0.815	1.227
External influence	0.193	0.05	0.179	2.573	0.011*	3	0.756	1.323
Gender (female = 1)	0.152	0.092	0.098	1.652	0.099	-	0.921	1.086
School type (public = 1)	0.103	0.090	0.068	1.144	0.253	-	0.894	1.119
R^2^ = 0.412	F = 23.756*							

***, **, and * represent significance levels of 1, 5, and 10%, respectively.

### 4.2 Person matrix of variables

This study carried out a systematic analysis of the correlations and significance among all variables, with a focus on presenting the correlation coefficients and significance levels between key demographic variables (e.g., household income, gender, school type) and core variables (self-cognition, psychological construction, external influences, anxiety performance). For instance, the correlation coefficient between household income and self-perception was r = − 0.23 (*p* < 0.01). The matrix results in Table 24 demonstrated a negative correlation between monthly household income and self-perception (r = − 0.23, *p* < 0.01), indicating that students from high-income households exhibited lower self-perceived anxiety, which was in line with the conclusion in Table 17.

**Table 24:** Pearson correlation matrix of all variables (r values, *p < 0.05, **p < 0.01).

Variables	Self-cognition	Psychological construction	Psychological construction	Anxiety performance	Monthly household income	Internship experience	Gender	School type
Self-cognition	1	0.42**	0.35**	0.58**	−0.23**	−0.19*	−0.12	−0.21**
Psychological construction	0.42**	1	0.31**	0.63**	−0.18*	−0.17*	−0.15*	−0.16*
External influence	0.35**	0.31**	1	0.49**	−0.15	−0.21**	−0.09	−0.14
Anxiety performance	0.58**	0.63**	0.49**	1	−0.20**	−0.24**	−0.16*	−0.18*
Monthly household income	−0.23**	−0.18*	−0.15	−0.20**	1	0.16*	0.08	0.13
Internship experience	−0.19*	−0.17*	−0.21**	−0.24**	0.16*	1	0.05	0.11
Gender (female = 1)	−0.12	−0.15*	−0.09	−0.16*	0.08	0.05	1	0.07
School type (public = 1)	−0.21**	−0.16*	−0.14	−0.18*	0.13	0.11	0.07	1

Internship experience exhibited a negative correlation with anxiety manifestations (r = − 0.24, *p* < 0.01), validating the finding that relevant internships alleviate anxiety, which was consistent with the observation presented in Table 17. Psychological construction demonstrated a strong positive correlation with anxiety manifestations (r = 0.63, *p* < 0.01), suggesting that inadequate psychological preparation serves as a core factor contributing to anxiety.

Female students (coded as 1) exhibited a significant negative correlation with anxiety manifestations (r = − 0.16, *p* < 0.05), which validates their relatively higher anxiety levels and supports the conclusion presented in Section 4.1.2. Public schools (coded as 1) showed a significant negative correlation with self-perception (r = − 0.21, *p* < 0.01), suggesting that students in public schools had lower self-perceived anxiety, thus corroborating the finding in Table 10.

External factors (e.g., the pandemic and policies) exhibited a significant negative correlation with internship experience (r = − 0.21, *p* < 0.01), indicating that students with extensive internship experience are less susceptible to external environmental pressures. These findings further corroborate the differential influence of demographic variables on employment anxiety, offering data support for targeted interventions. All correlation analyses passed significance tests (*p* < 0.05 or *p* < 0.01), establishing the groundwork for subsequent regression analyses and mechanism exploration.

### 4.3 Causes of employment anxiety

The post-hoc analysis identified the “monthly household income ranging from 30,000 to 80,000” group and the “non-affiliated internship” group as critical sensitive populations prone to anxiety, thus providing targets for targeted interventions. Regression analysis demonstrated that the enhancement of self-cognition (e.g., skill improvement and the establishment of career confidence) was the most effective in alleviating anxiety, followed by psychological construction (e.g., adaptive training and expectation management). The findings support the self-efficacy theory, which posits that individuals’ belief in their capabilities (self-cognition) is the primary predictor of employment anxiety, validating the hypothesis that “internal factors play a dominant role in the formation of anxiety.” Therefore, the causes of employment anxiety among these pre-service Chinese language teachers can be attributed to the following main factors:

#### 4.3.1 Students lack self-confidence

Job interviews are inherently marked by uncertainty, with outcomes that are challenging to forecast, often leading students to perceive unfavorable results. This phenomenon makes interview anxiety a salient characteristic of employment-related anxiety. A widespread problem among contemporary college students is the deficiency in self-confidence. The survey reveals that 20% of the respondents consider themselves lacking in distinctive skills, and 14.1% assert that they have no proficiency in foreign languages. When faced with academic or career challenges arising from insufficient personal qualifications, students may lose confidence in securing relevant employment, question their employability, and subsequently experience anxiety, indecision, or even opt to abandon their pursuits.

Students characterized by low self-cognition, such as those who assess their teaching skills as substandard, encounter anxiety arising from negative evaluations of their professional competence.

In Tables 1, 9, 23, within the self-cognition dimension, the mean scores of ZW3 (insufficient teaching skills, M = 2.93) and ZW4 (inability to distinguish oneself, M = 3.05) were notably lower than the mid-point of the scale (3.0). This indicates that approximately 50% of students encounter self-doubt concerning their competence. Moreover, this dimension demonstrated the strongest regression coefficient with employment anxiety (*β* = 0.382, *p* < 0.001).

A survey conducted by Lu (2009) among senior students majoring in education indicated that a higher level of job-hunting self-confidence is associated with a reduced number of obstacles. In the context of English education in Taiwan, China, students have generally achieved proficiency in basic language skills. Nevertheless, the survey results disclose that students exhibit a lack of confidence in their linguistic capabilities. Some students opine that mastering only one foreign language is insufficient for competitive purposes and even perceive themselves as linguistically deficient.

A survey on internship participation revealed that 43.57% of students had neither major-related internships nor non-major-related internships. These students exhibited significantly higher degrees of anxiety compared to those who had participated in major-related internships. Data imply that students who have completed major-related teaching internships, regardless of whether they are from public or private universities, gain valuable experience, boost their confidence, and face fewer challenges during the job-seeking process. However, the majority of students lacking such experience regard themselves as less competitive, experience a decrease in self-confidence, and subsequently develop job-seeking anxiety.

Within the psychological construction dimension, the elements of “salary expectations” and “parental expectations” indirectly suggest that heightened expectations may exacerbate anxiety. Table 8 indicates that 48.55% of students expect a monthly salary in the range of NT$30,000 to 80,000, while only 17.43% believe they can achieve a salary of NT$80,000–150,000, demonstrating a considerable discrepancy between expectations and reality. When integrated with Tables 1, 9, 23, items XL2 (perceiving that post-employment salary will fall short of expectations, M = 2.84), XL5 (believing that current education cannot ensure satisfactory employment, M = 2.77), and XL3 (work not meeting parental expectations, M = 3.03) demonstrate that students generally encounter pressure arising from family expectations. This item shows a significant positive correlation with anxiety performance (*β* = 0.251, *p* < 0.01).

#### 4.3.2 Impact of family conditions

According to Wang and Jiang (2022), the family environment, parent-child relationship, economic conditions, and parental expectations are significant influencing factors of employment anxiety among college students. Students’ academic and career choices are related to themselves, their families, the environment, and their schools, among which family factors are the most crucial. Parents provide both tangible and intangible social resources for their children.

This survey examined family composition and monthly household income. Family members act as implicit indicators of family dynamics. Students with multiple siblings are more likely to possess a more comprehensive self-perception and stronger independence. In the dimensions of self-perception and anxiety manifestation, students from two-child families score below the mean. This is due to the competitive mindset of elder siblings and the absence of positive guidance, which often result in self-doubt and self-denial. Monthly household income serves as a visible indicator. Students from financially disadvantaged families exhibit prominent employment anxiety, whereas middle-class students are more susceptible to external influences. Students from families with a monthly income exceeding NT$150,000 report the least anxiety, as they benefit from material support, a diverse range of interests, and confidence in their capabilities.

A substantial number of parents offer guidance and career-planning advice when their children apply to universities. Students with pre-established academic pathways demonstrate a stronger sense of competition and purposefully enrich their university experience. In contrast, those who only begin to consider job-hunting near graduation are ill-prepared for social integration, and their anxiety levels fluctuate in line with environmental changes.

#### 4.3.3 The school lacks guidance

Newly graduated students entering the society undergo a transition from the campus environment to the real-world context. Due to public health concerns from 2020 to 2022, a substantial number of graduates in 2023 and 2024 stayed on campus, often experiencing a sense of confusion and helplessness. The survey findings reveal that 63.9% of students had developed career plans before starting their college studies, while 33.61% had formulated such plans prior to enrollment. A significant difference in students’ self-perception was identified (*p* < 0.05), indicating that universities’ career planning courses and employment support are insufficient. An examination of academic curricula shows that most institutions consider career planning courses as a low-priority discipline. Interviews with students indicated that teachers did not attach sufficient importance to this subject, with classes mainly relying on textbook content and having minimal interaction. Students reported a lack of adequate career guidance and continuity throughout their academic period, and schools had limited practical links with the social realm. Teachers failed to provide personalized career planning, leaving students vulnerable to career risks, setbacks in job-seeking, and anxiety stemming from a lack of confidence in their career planning.

The fundamental cause of career anxiety among college students resides in the discrepancy between their professional aspirations and the actual circumstances. Freshmen and sophomores, having recently emerged from the high-pressure milieu of high school, frequently lack career planning during their vibrant university years, adhering to the conviction that success will transpire spontaneously without proactive endeavors. It is essential for universities to provide career planning courses, where instructors ought to set an exemplary standard to aid students in establishing career foundations, achieving their objectives, and smoothly transitioning from the campus to the workplace.

#### 4.3.4 Lack of policy support

The official website of the relevant educational authority in Taiwan, China, reveals that following the implementation of the New Southbound Policy, the region has introduced a series of measures to strengthen Chinese language education. These measures include providing subsidies to attract overseas Chinese language educators for professional development, offering scholarships to encourage students to engage in Chinese language learning, establishing partnerships with Western institutions for promotional activities, awarding summer scholarships to university students, and launching Chinese Language Navigation Programs at prominent overseas universities. However, these incentives are mainly aimed at non-native Chinese speakers, and the subsidies are only available to overseas teaching personnel. A survey shows that only 26.14% of students are aware of these policies and employment opportunities, while 33.2% are completely uninformed, which highlights the insufficiency of public awareness campaigns.

A multitude of universities in Taiwan, China, have established Chinese language and literature teaching departments, yielding a significant number of graduates. A notable proportion of these graduates harbor aspirations to engage in teaching overseas. Nevertheless, there exists a scarcity of overseas institutions in collaboration with those in Taiwan, China, and the demand for teaching positions is limited. The living conditions for overseas teaching are less advantageous in comparison to those in Taiwan, China, accompanied by relatively meager subsidies. Students often encounter trepidation and anxiety when contemplating teaching in overseas environments.

Regarding teaching positions in Taiwan, China, the Chinese Language Teaching Department implements non-traditional teaching methods, and there are substantial discrepancies in professional training programs across different universities. Graduates from this department are not qualified to acquire teaching credentials for public schools. Although some international students have established themselves in southern Taiwan, China, the northern region provides more employment opportunities but also experiences intense competition, inducing significant anxiety among students. Those pursuing specialized teaching positions frequently choose international schools or private tutoring institutions. Nevertheless, teaching at private institutions may entail involvement in recruitment and promotional activities, which could potentially compromise teaching quality.

### 4.4 Recommendations for alleviating students’ employment anxiety

Students are anticipated to actively elevate their self-worth. They ought to separate emotions from reality, assume an objective perspective towards career development, and courageously accept failures while learning from interview setbacks. Anxiety generally stems from internal factors. Through interview experiences, students need to reinforce their self-efficacy and foster a positive mindset. Majors in Chinese Language Education should leverage failure as a stepping-stone to become more proficient educators. In their spare time, participating in group activities and foreign-language clubs can significantly enhance language logic and communication skills. Students without remarkable talents should observe the strengths of others and seek advice to precisely assess their capabilities. When faced with career-planning difficulties or negative emotions, students should not hesitate but engage in collective ideation with peers, consult mentors, and adopt others’ strategies to improve their teaching methods and lifestyles. Although the majority of students have pre-employment plans, some neglect interpersonal relationships during their academic years and lack in-depth self-awareness in career planning. Access to first-hand employment information is of great importance, as basic communication skills may result in missed opportunities due to information disparities. Students actively involved in campus activities often encounter interpersonal challenges, which contributes to the development of versatile skills crucial for making well-informed career decisions. Considering the balanced economic development and intense workplace competition in Taiwan, China, students are required to assiduously enhance their competitiveness for better career prospects.

Family members typically provide reciprocal support. Parental education and support constitute the cornerstone of children’s lives. Nurturing children’s comprehension and satisfaction of their needs is the fundamental responsibility of parents, and their behavioral patterns are reflective of their children’s development. Research conducted by Su et al. (2013) demonstrated that college students place significant emphasis on family affection and interactive relationships. As architects of the family atmosphere, parents should actively interact with their children to establish a supportive and encouraging family environment. Wu (2001), through interviews with college students throughout Taiwan, discovered that students experiencing financial hardships perceived unhappiness, whereas those with stable finances considered financial factors to be non-significant contributors to employment anxiety. This implies that family financial support can mitigate college students’ employment anxiety. Furthermore, students with a greater number of siblings tend to express their needs more comprehensively. Every individual encounters challenges at various stages, and family understanding, attentive listening, tolerance, and encouragement are of utmost importance. When college students confront employment difficulties, support from parents and siblings in the form of networking or financial aid can alleviate job-seeking pressure.

The university has further intensified career-guidance measures. When organizing campus activities, the institution can enforce structured requirements to stimulate student participation. The administrative department should actively improve educational training by establishing career-counseling centers. Specialized consultations in these centers can assist in alleviating students’ employment-related anxiety and preventing the adverse effects of such stress on health. Faculty members can modify the content of career-planning courses, integrating STEAM pedagogy to devise diversified instruction, which can demonstrate and train students’ observational, imaginative, creative, and logical-thinking capabilities, thus enhancing comprehensive teaching competencies and enabling students to apply knowledge in practical situations. Career-guidance programs also foster students’ foreign-language proficiency and promote talent development to enhance their competitiveness in the workplace. The university maintains a “Student Career Column” to gather feedback and promptly revise career-guidance strategies. Additionally, Chinese-language departments regularly organize “Chinese Language Teacher Career Forums,” inviting experienced educators to give lectures and offer consultations, and adopting a mentorship approach to optimize talent cultivation.

It is imperative to enhance social support measures. Environmental factors can readily induce anxiety. Interviews with graduates from Chinese Language Education programs indicate that despite the active development of Chinese-language education in Taiwan, China, challenges such as limited employment opportunities, frequent issues in overseas employment, and substantial disparities in subsidies between local and international students remain crucial. These problems mirror the scarcity of local teaching resources, imperfect mechanisms, the absence of a comprehensive education system, and insufficient teacher benefits in Taiwan, China, which give rise to employment anxiety and potential brain drain.

To tackle this, society should strengthen communication, actively promote initiatives in Chinese-language education, and offer more abundant teaching resources. Project teams should be established to integrate and promote Chinese-language education overseas, construct a teaching system, and create more opportunities for teacher development. Welfare subsidies for educational institutions and personnel should be increased to prevent talent loss, attract overseas elites to Taiwan, China, and improve teacher-training and support systems. Diversified online Chinese courses should be developed, online teaching and learning should be advanced, multi-platform resources should be integrated, and the teaching-system mechanisms should be refined.

## 5 Conclusion

This research utilized a theoretical framework to carry out a comprehensive exploration of the factors and mechanisms affecting employment anxiety among Chinese language teaching majors in four Taiwanese universities (the 2023–2024 cohort) through a questionnaire survey (*N* = 241) and interviews (*N* = 16). The primary findings are presented as follows:

Students in Chinese language departments exhibited a moderate degree of employment anxiety. This anxiety was primarily driven by self-perceived factors (e.g., skill inadequacies) and external factors (e.g., the effects of the pandemic). Furthermore, anxiety-related behaviors further intensified the anxiety levels.

In the realm of psychological construction dimensions, female students manifested significantly higher levels of anxiety than male students (*p* < 0.05). Students from public schools reported a greater degree of self-perceived anxiety in comparison to those from private schools (*p* < 0.01). Students hailing from families with a monthly income within the range of NT$80,000 to NT$150,000 recorded the highest levels of anxiety.

Students who engaged in industry-related internships exhibited significantly lower levels of anxiety compared to those without internships or those with non-related internships (M = 2.77). Students possessing English proficiency along with skills in a minor language manifested the lowest anxiety levels. Merely 26.14% of the students were aware of industry policies, and the overseas employment group presented the highest anxiety levels. The combined explanatory capacity of self-cognition, psychological construction, and external influence on employment anxiety reached 75.25%. Self-efficacy and perceived policy support functioned as crucial moderating variables.

Through systematic exploration and empirical analysis, this study provides theoretical foundations and practical methods for alleviating employment anxiety among students majoring in Chinese language teaching in Taiwan, China. As the first research focusing on this specific group, it overcomes the limitations of existing studies that primarily target students from the Chinese mainland or international students. The study systematically reveals unique contributing factors. For example, students from families with incomes ranging from NT$80,000 to NT$150,000 exhibited stronger anxiety, and students in public schools had significantly higher self-cognition anxiety than those in private schools. This offers regional empirical support for the application of employment anxiety theory in Chinese language education.

Based on the theory of planned behavior and self-efficacy theory, the study clarifies the interaction mechanism of “internal factors (self-cognition)-external factors (policy support)-anxiety manifestation”. Empirical results show that the interaction between internal factors (such as self-doubt regarding skills) and external factors (such as the influence of the pandemic) accounts for 75.25% of the variance in anxiety. By integrating multi-dimensional influence mechanisms, this research enriches the theoretical framework of “environment-individual-behavior” interactions.

This study offers evidence-based interventions to relieve students’ employment-related anxiety. In light of findings such as “higher psychological construction anxiety among female students” and “anxiety reduction through targeted internships”, it guides students to alleviate anxiety and improve employability via skill development and internship feedback journals. Taking into account issues such as “textbook-centered teaching” and “lack of practical integration” in school career planning courses, the study suggests that universities introduce Cross-Strait Chinese Language Teaching Comparison Modules and Digital Teaching Skills Workshops, and establish University-Enterprise Collaborative Internship Networks to optimize the employment support system. Concerning the challenges encountered by Taiwanese Chinese language teachers, including “lack of public-sector positions” and “insufficient overseas job opportunities”, policy suggestions such as “salary subsidies for overseas employment” are proposed to provide data-supported assistance for policy-making.

It serves to mitigate the issue of talent drain within the industry. Owing to the constraints of the survey, this research centers on four universities in Taiwan, China, offering partial perspectives on the employment anxiety of Chinese language teaching majors and failing to fully represent the broader Taiwanese situation. Future research ought to expand sample sizes, conduct in-depth mechanism analyses, and assess policy effectiveness. Potential research directions include broadening the scope to cover more Taiwanese institutions for a comparison of anxiety levels between pre-service Chinese teachers from the Chinese mainland and Hong Kong, thus revealing the impacts of regional policies. Conduct 1–3-year follow-up studies to analyze anxiety patterns specific to different career stages and long-term driving factors. Incorporate moderating variables such as social support and cultural adaptability to examine their mediating roles in the self-cognition-employment anxiety relationship, and explore how career identity mediates the internship experience-anxiety relief pathway. Explore the potential influence of AI through interdisciplinary approaches.

## Data Availability

The original contributions presented in the study are included in the article/supplementary material, further inquiries can be directed to the corresponding author.
